# Editorial: Group norms and moral development: Reasoning and cognition across the lifespan

**DOI:** 10.3389/fpsyg.2022.1035999

**Published:** 2022-10-13

**Authors:** Clare Conry-Murray, Luke McGuire, Aline Hitti, Hanna Beißert

**Affiliations:** ^1^Department of Psychology, Saint Joseph's University, Philadelphia, PA, United States; ^2^Department of Psychology, University of Exeter, Exeter, United Kingdom; ^3^Department of Psychology, University of San Francisco, San Francisco, CA, United States; ^4^Department of Education and Human Development, DIPF | Leibniz Institute for Research and Information in Education, University of Konstanz, Konstanz, Germany

**Keywords:** moral development, group norms, intergroup attitudes, developmental psychology, culture

As intergroup interactions increase with global migration, it is important to understand how youth consider group interests in relation to justice and equal treatment. The current Research Topic aims to examine the interplay between morality and group processes throughout development as they bear on youths' decisions about inclusion, when to challenge prejudice, and how to distribute valued resources. Extensive research has documented the early emergence of youth's concerns for fairness, equity, and justice (e.g., see work by e.g., Turiel, 2008; Killen and Smetana, 2013), and growing awareness of group memberships and norms (e.g., Aboud, 2003).

The current Research Topic brings together a set of papers, using a broad variety of different methodological approaches, and covering three main themes: (1) To what degree are children's moral judgments affected by social groups across different contexts? (2) How do children and adolescents differ in ways that may provide insight into development? (3) What possible mechanisms explain judgments of groups? We discuss each of these briefly below, citing the literature in this collection where readers can find more evidence and discussion.

## Are moral judgments affected by social groups across different contexts?

A central theme that emerges from this Research Topic is the question of whether in-group bias varies across contexts. While one may expect that children show preference for their in-group across contexts (Nesdale and Flesser, 2001; Aboud, 2003), this set of studies demonstrated the contextual nature of in-group bias, and the role that group norms play in determining when it is acceptable to favor one's in-group.

In early childhood, when preferences are pitted against group membership, Yang and Park found that group membership trumped preferences; thus children allocated more resources to one's in-group member even though an out-group member liked the same thing as they did. These decisions were driven by underlying beliefs about in-group loyalty and obligation to one's group. In other contexts, children showed a more nuanced understanding of group membership by demonstrating a sensitivity to group status. Yee et al. showed that children demonstrated an understanding that high wealth groups may hold more in-group biased norms compared to popular groups. Yet in another context, Yuly-Youngblood et al. showed that children do not use group membership to judge an act of physical aggression, particularly when it is intentional, but show bias when judging other forms of aggression (e.g., relational).

Moral concerns for harm appear to trump in-group membership in terms of judgments, but do they also affect behavior? When allocating resources to different groups, Corbit et al. demonstrated older children come to understand that it is contextually inappropriate to favor one's in-group, instead discarding resources to ensure fairness for both in-group and out-group members.

## Developmental differences in reasoning about groups

Many studies have shown that older children and especially adolescents are increasingly able to coordinate multiple factors in their moral judgments (Killen and Smetana, 2013). The current collection of studies shows that adolescents are more likely than children to consider moral consequences in intergroup situations. In particular, Gönültaş et al. found adolescents were more approving of bystanders who challenged exclusion of immigrant peers, even when in-group members espoused exclusive attitudes. Additionally, German adolescents in Beißert and Mulvey showed inclusive orientations toward Syrian refugees despite their expectations that in-groups would be less inclusive.

Additionally, Farooq et al. examined evaluations of peer group members who misinform and breach moral principles of honesty. While both children and adolescents evaluated an in-group misinformer more positively than an out-group misinformer, adolescents, compared to children, understood that a misinformer may have more positive intentions. Coupled with the findings in Yuly-Youngblood et al., these findings show indications of development. In more straightforward contexts (e.g., physical aggression), children are capable of balancing their in-group preference with competing contextual and moral information, whereas in other more complex settings (e.g., misinformation) it is not until adolescence that youth can use their knowledge of the setting to inform their judgments.

Developmental differences were also found in help-seeking behaviors, and these were related to underlying beliefs about trustworthiness and loyalty. For example, Yüksel et al. found that children were more likely to seek help from teachers after witnessing someone being excluded while adolescents were more likely to seek help from peers. Their reasons for this help-seeking behavior differed, highlighting the importance of investigating participants' reasoning.

## Mechanisms for group influence on moral judgment

What factors explain judgments about groups' influence on moral judgment? While most agree that moral judgments involve a consideration of the impact of the protagonist's behavior on others (e.g., Piaget, 1932), the current collection also points to cognitive processes such as considering other people's emotions (Stowe et al.), intent (Yuly-Youngblood et al.), and beliefs. For example, Stowe et al. demonstrated that emotional cues are used to make moral judgments when children, as young as 5 years, recognize that someone will feel bad about receiving less stickers they are more likely to judge the distribution as unfair.

Many social situations also require an understanding of others' minds (i.e., Theory of Mind), an ability often acquired with age. For instance, Gönültaş and Mulvey found participants in middle school were more likely to attribute mental states to their in-group members, while high school participants were just as likely to attribute mental states to in-group and out-group members.

Finally, group membership and social norms may play a role by serving as sources of information about intent or emotional response. For example, Farooq et al. found group membership informed attributions of intentions, where children believe that an out-group member was intentionally misinforming others more than an in-group member.

## Conclusion

The studies in this collection represent a globally diverse sample of children and adolescents (e.g., China, Germany, Turkey, U.K., U.S.A), and they indicate that across cultures, children consider moral principles, while they are also influenced by group membership. The findings from this collection suggest that concepts of group loyalty, often studied in adolescence, may impact children as well, through their understanding of intentions, beliefs, and emotions.

## Author contributions

All authors listed have made a substantial, direct, and intellectual contribution to the work and approved it for publication.

## Conflict of interest

The authors declare that the research was conducted in the absence of any commercial or financial relationships that could be construed as a potential conflict of interest.

## Publisher's note

All claims expressed in this article are solely those of the authors and do not necessarily represent those of their affiliated organizations, or those of the publisher, the editors and the reviewers. Any product that may be evaluated in this article, or claim that may be made by its manufacturer, is not guaranteed or endorsed by the publisher.
